# Controlling Electronic Devices with Brain Rhythms/Electrical Activity Using Artificial Neural Network (ANN)

**DOI:** 10.3390/bioengineering6020046

**Published:** 2019-05-17

**Authors:** Yar Muhammad, Daniil Vaino

**Affiliations:** 1 Narva College, University of Tartu, Narva 20307, Estonia; 2 Narva Pahklimae Gymnasium, Narva 20605, Estonia; Daniil.Vaino@gmail.com

**Keywords:** EEG (electroencephalography), ANN (artificial neural network), BCI (brain-computer interface), BFB (biofeedback), bitronics, Arduino, machine learning

## Abstract

The purpose of this research study was to explore the possibility to develop a brain-computer interface (BCI). The main objective was that the BCI should be able to recognize brain activity. BCI is an emerging technology which focuses on communication between software and hardware and permitting the use of brain activity to control electronic devices, such as wheelchairs, computers and robots. The interface was developed, and consists of EEG Bitronics, Arduino and a computer; moreover, two versions of the BCIANNET software were developed to be used with this hardware. This BCI used artificial neural network (ANN) as a main processing method, with the Butterworth filter used as the data pre-processing algorithm for ANN. Twelve subjects were measured to collect the datasets. Tasks were given to subjects to stimulate brain activity. The purpose of the experiments was to test and confirm the performance of the developed software. The aim of the software was to separate important rhythms such as alpha, beta, gamma and delta from other EEG signals. As a result, this study showed that the Levenberg–Marquardt algorithm is the best compared with the backpropagation, resilient backpropagation, and error correction algorithms. The final developed version of the software is an effective tool for research in the field of BCI. The study showed that using the Levenberg–Marquardt learning algorithm gave an accuracy of prediction around 60% on the testing dataset.

## 1. Introduction

This research study focuses on measuring the performance of artificial neural network (ANN) and the developing of brain computer interface (BCI). In order to acquire the electroencephalography (EEG) datasets, this study has investigated EEG sensor capabilities for using it as a new interface in order to receive information and control any devices, such as wheelchairs, computers and robots. This is currently a highly relevant and important topic of focus.

The most popular devices for computer input are keyboard, mouse and screen-sensor. The human brain generates an electrical field/waveform. This kind of input could be used to control computers or other devices. This study focuses on creating an interface between a human and a computer and controlling the computer through human brain activities. This research is focused on investigating and monitoring electroencephalography (EEG), as well as analyzing and understanding the recorded electrical activity of the brain in order to use this knowledge to control computers or any possible devices. The main challenges in the development of BCI are acquiring the EEG data, analyzing the data in a real-time environment and giving real-time feedback to the subject. In this research, several disciplines are involved, such as electronics, biomedical engineering, computer science, mathematics, physics and programming. To understand what makes it possible to understand brain activity, it is necessary to pay attention to, and to understand, certain features and characteristics of the brain’s activities.

There are 86 to 100 billion neurons in the brain [1]. According to research, all of them are united in one structure by means of synapses. The synapse-place of contact between two neurons serves to transmit a signal (inhibitory or exciting) from a neuron to a neuron. Synapses are chemical (used for signal transmission neurotransmitters) and electrical (the signal passes from the surface of one neuron directly to the surface of the other by means of charged particles). The electric potential moves along the neurons. Thus, both these and other synapses use electricity to transmit a signal. When the neurons are activated, the neurons transmit the signal over the dendrites and through the synapse to the axons of other neurons. To activate the neuron, it is necessary to excite it [2]. A neuron generates a signal, which is transmitted to other neurons if its own excitation level reaches a certain value. This level is determined by the sum of all incoming excitation and decelerating signals [1].

Electroencephalography (EEG) is a complicated summation of the potentials of many neurons working independently. In the case of significant temporal synchronization of the activity of neurons, there will be a strong deviation of the potential that is referred to as EEG rhythms. The total EEG is made up of the sum of all of the recorded rhythms [1].

There are several types of waves regarding the neuron signals. The main waves are alpha (8–13 Hz), beta (16–31 Hz), gamma (32–100 Hz) and delta (0.5–3 Hz). The alpha and beta waves have the greatest possibility to change the amplitude (quick response) of the signal. The goal of meditation is to increase the alpha rhythm activity. Beta rhythm indicates higher cognitive processes and it increases when someone focuses on particular things [1].

**Figure 1:**
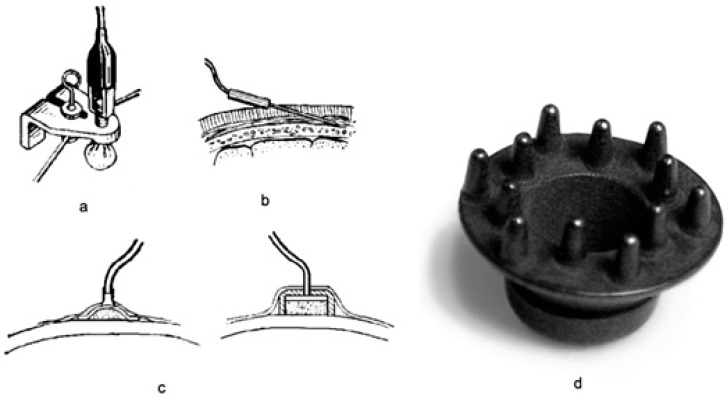
Types of electrodes and how to configure them to acquire useful signals. (**a**) bridge electrode; (**b**) needle electrode; (**c**) cup electrode; (**d**) dry electrode.

Technically, the brain-computer interface (BCI) starts to read the EEG waveform in order to obtain data. It is necessary to transfer the electric potential with the help of an electrode in order to amplify the signal, wherein afterwards the signal is converted with the help of an analogue-to-digital converter [1]. There are several types of electrodes, such as bridge electrodes, needle, cup and dry electrodes [1], that can be employed for this purpose. These are depicted in Figure 1.

(a) Bridge electrode: This electrode is composed of a metal rod with a holder which is covered at one end with a hygroscopic material and impregnated with a solution of sodium chloride. From the opposite end, the tap leads off. Bridge electrodes consist of a porous material (mostly sponge, cotton or wool); they can be attached to a special cap/helmet or soaked with an electrolyte.

(b) Needle electrode: This electrode is absorbed into the skin. It is mostly used during surgical operations to monitor the central nervous system. Needle electrodes do not require an electrolyte. They provide good contact due to low resistance which contributes to the flow of electrical potential.

(c) Cup electrode: This electrode is made of metal and has the shape of a hemisphere. It contacts an electrolyte, which is located on the human skin. Cup electrodes are, in fact, the metal plates that are placed on the cap and an electrolyte is applied underneath them. 

(d) Dry electrode: This electrode is made in the form of a brush or a plate with several pins, which is necessary for good contact with the skin. It is used without electrolytic solutions or pastes. Dry electrodes are metal plates with protuberances that are attached to the skin through the hair. They require a tight attachment to the skin of the head in order to ensure good contact [1].

In this study, dry electrodes were used for the experiments, since they do not require electrolyte application on the head surface. This process highly simplifies the experiments, however, these types of electrodes requires the use of an elastic bandana to tighten the attachment of the electrodes to the skin. Dry electrodes have high resistance compared with bridge, cup or needle electrodes, which may increase the level of interference, but this must be eliminated by means of a differential amplifier (an electronic amplifier with two inputs, with the output equal to the difference of the input voltages multiplied by a constant).

**Figure 2:**
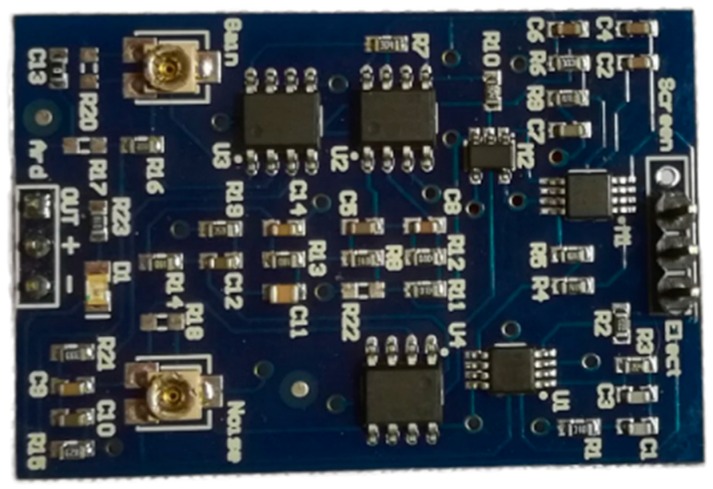
The Bitronics EEG module.

Currently, the research on BCI is becoming promising and popular. The technologies are continuously developing, and now possibilities of the implementation of portable encephalographs have become available, such as Bitronics (Figure 2), the Emotiv EPOC+ and the NeuroSky ThinkGear ASIC module (TGAM).

The Bitrionics EEG is a single-channel EEG compatible Arduino module; its big advantage is that it is able to make use of dry electrodes and can use two electrodes simultaneously as a differential input, which effectively eliminates interference. [1].

Currently, computers are becoming increasingly more powerful, which allows us to process the signal directly on a computer without resorting to the help of extraneous computing power (using portable devices such as laptops or mobile phones). Moreover, significant progress has occurred in the field of algorithms. Machine learning and neural networks are a fundamentally new way of data processing which is being actively used to effectively solve a huge number of tasks and which can possibly help to implement BCI. Therefore, brain electrical activity can be detected and used as an input signal, and can be compared with modelled waveform for each activity to control devices and computers [3]. 

This paper is structured as follows: Section 1 provides an introduction to the research; Section 2 presents the related work of EEG devices, algorithms and the central nervous system (CNS); Section 3 discusses the libraries which are used for the implementation of machine learning, hardware configuration and conduct the experiments; Section 4 demonstrates the results which have been obtained in the present study; finally, Section 5 concludes with a summary of the main contributions of the paper and possibilities for future work. 

## 2. Literature Overview

The literature survey is divided into three formal groups: the first is popular articles, the second is commercial publications and the third is scientific works and articles. 

The literature survey started with the article by Roman Fishman published in Popular Mechanics Magazine [4]. The description of EEG equipment was given based on a Bitronics Lab product. It describes how people managed the EEG Bitronics Lab, which measured the amount of alpha rhythm without any problems to control the speed of movement of a toy car. This non-complex experiment shows the use of EEG with biofeedback (BFB) to manage the interface. In this experiment, the researchers used an analogue EEG sensor which made it possible to record the potential value on the cerebral cortex with a very high frequency (writing speed depends on the microcontroller. Arduino, the board used in this experiment, is able to read the signal up to 10,000 times per second. It is a high index for the EEG). In addition, there are various additional sensors such as the skin-galvanic reaction sensor, electrocardiogram sensors and electromyograms, which are included in the kit.

There are different so-called “neural sets” now available. Some are becoming popular, such as NeuroSky, Emotiv EPOC+ and Bitronics EEG. The website neuromatix.pro regularly publishes articles on the topic of EEG. In April 2015, the article “Neurointerfaces of the consumer class, features and applications” was published [5]. In the article, the essential part is a review of portable EEG headsets and interfaces. Most of the devices described in this article are based on the principle of EEG—they measure the amount of alpha rhythm and are controlled through the relaxation/concentration of attention which allows us to observe the process of meditation. In addition, the article covers the history of the origin and development of the EEG, principle of the EEG and the work done with this signal. NeuroSky is a company that has been engaged in the development of portable EEG equipment since 1999. The company has released several different headsets for monitoring the state of the brain, as well as the TGAM module for free development on EEG-based systems. There are two sensors in their device; the device provides output of the potential difference between the frontal part of the head and the ear as soon as there is no brain activity. Their systems allow us to manage different toys, such as a helicopter, a lamp and other devices, and play some relaxing and concentrating games. Emotiv EPOC+ is a similar wireless EEG headset. However, it has a number of differences: 14 sensors instead of one, a closed source code and encrypted data. The Emotive EPOC+ shows only the result of the analysis; to have the raw data signal from this device you will have to pay extra. On the headset, in addition to EEG sensors, there are gyroscopes and accelerometers. There is a number of programs for signal processing in the complete set, along with the equipment.

In the article “The use of EEG bio-management technologies for the correction of the psychoemotional state of children” [6], some experimental results are given which describe biofeedback technologies that were used for the correction of the condition of children experiencing communication difficulties. The amplitude of the alpha rhythm, the ratio of the alpha and theta amplitude of the rhythms, as well as the amplitude of the remaining rhythms were estimated. BFB was carried out through sound (white noise) and visual (figures on the monitor) contact [6]. In general, the ability of the subjects to deliberately maintain the required level of amplitude, as well as the overall improvement in the central nervous system (CNS) state was noted. Anxiety, frustration and feelings of inferiority decreased, and improvement in indicators of voluntary attention were noticed. The work was carried out on the basis of the Taurida National University [7]. 

The article “Possible mechanisms of the action of biological feedback on the electroencephalogram” [8] discusses the possibility of BFB with the EEG method. The possibility of a purposeful influence on the system is described. As experiments, the BFB systems are used to control the enhancement of alpha activity and to reduce beta and theta activity. The article discusses the theoretical neuro-bio-physical principles of EEG-BFB. It also describes that regardless of the type of the EEG-BFB (alpha/beta/gamma rhythm amplitudes), the CNS becomes more stable. The work was also carried out on the basis of the Taurida National University [7].

The details of the operations performed on the EEG signal for analyzing the state of the brain are discussed in the article “Non-linear analysis of dynamic systems and its application for EEG signals processing” [9].

**Figure 3:**
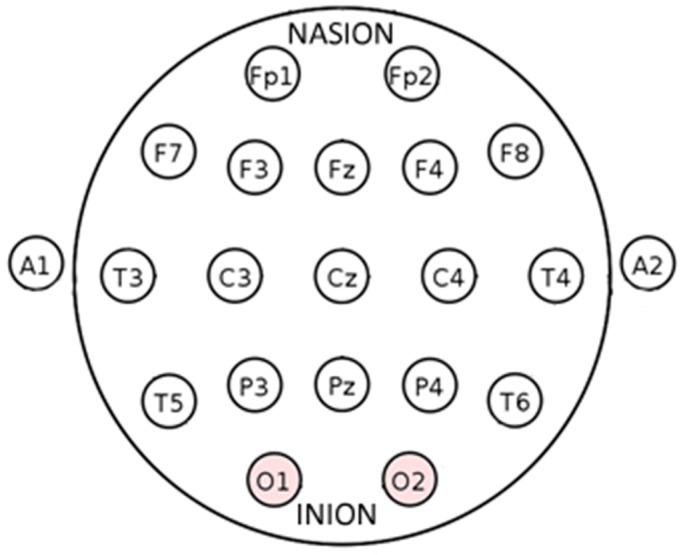
Electrode locations of International 10–20 system for EEG (electroencephalography) recording. Fp1, Fp2, F7, …, T6, O1, O2 are points for electrode placement.

The research work “Pattern recognition for non-invasive EEG-based BCI” [10] describes the principles of synapses, the physics of the appearance of electrical potential on the surface of the brain and the principles of the EEG. It describes the arrangement of the 10–20 electrode system (Figure 3), discusses the main rhythms of the brain and talks about the two devices used, which were Emotiv EPOC+ and Biosemi ActiveTwo [10]. The electrode International 10–20 location system is used in the study to acquire the EEG signals.

Bitronics has two built-in programs. One is for Windows operating systems and the second is sketch. Sketch is used for Arduino. Sketch periodically takes readings from two analogue ports and sends the value to the computer in the form of lines, adding “A1” and “A2” to them, depending on which analogue port voltage value is sent. 

The work of Fourier transforms is explained in the same way. The essence of the experiments is the use of machine learning algorithms to analyze the thoughts of the subject. As for the input data, an EEG signal was used, and was transformed into a frequency spectrum by means of Fourier transforms. The machine learning algorithms OneR, J4.8 (which is the development of the C4.5 algorithm [10]), Naïve–Bayes and self-organizing map of Kohonen (SOM) and Waikato Environment for Knowledge Analysis (WEKA) implementation of support vector machines (SVM) were used [10].

During the experiments, the data were extracted from two different EEG devices. The first one was inexpensive and aimed for a wide range of users Emotiv EPOC+ with 14 electrodes, and the second one was a high-end Biosemi ActiveTwo with 32 electrodes. Two experiments were taken and results of both experiments were analyzed. In the first experiment, the best result was achieved using the Naïve–Bayes algorithm. The trained model was able to classify brain signals and mentioned if the subject was thinking left or right with 84% accuracy. In the second experiment, the best result of 52.4% accuracy was achieved with Sequential Minimal Optimization (SMO) algorithm. The major accuracy improvements were achieved by enlarging the size of the window and sampling frequency. The best results of 52.4% show that by only looking at the subject’s brain signal, the model was able to correctly detect the kind of half of the figures shown to the subject. These results show that future work in this direction can yield even better outcomes.

The research article “Adaptive Interactive Learning: A Novel Approach to Training Brain-Computer Interface Systems” [3] is very similar to [10]. It discusses the principles of synapses, the brain electrical potential, the EEG system and machine learning. Nevertheless, several other machine learning algorithms were considered, such as SVM, Naïve–Bayes classifier, Decision tree classifier, SOM, K-means, performance evaluation and PCA (principal component analysis) [3]. The work mentions and indirectly uses BFB [3], but it does not focus on BFB.

However, distinguishing mental states of a human is a challenging task and machine learning alone is not enough to solve the problem. The acceptable level of performance can be achieved after a long training process, during which the human learns how to produce suitable mental states and the machine creates a model which is able to classify the signal.

The research work “Control a robot via VEP using Emotiv EPOC+” [11] also studies the mechanism of the emergence of potential on neurons. The experiments use VEP (visually evoked potentials), specifically SSVEP (steady state visually evoked potentials) technology instead of machine learning. The work describes an SSVEP-based BCI implemented as an empirical part of this work. One possible usage of a BCI that efficiently implements a communication channel between the brain and an external device would be helpful for disabled people to control the devices that currently require pushing buttons, such as electric wheelchairs. The BCI is implemented as a part of this work [11], and uses widely known PSDA (power spectrum density analysis) and CCA (canonical correlation analysis) feature extraction methods and introduces a new way to combine these methods. The application was tested only superficially and the following results were obtained: 2.61 ± 0.28 s target detection time, 85.81 ± 6.39% accuracy and 27.73 ± 5.11 bits/min ITR (information transfer rate). The SSVEP-based BCI is available and is open-source. The software, written in Python 2.7, has a graphical user interface (GUI) and uses an inexpensive EEG device called Emotiv EPOC+. The BCI requires only a computer and Emotiv EPOC+. Other than that, no additional hardware is needed. Different EEG devices could be used after making some modification in the code.

When developing the experiments, the data was obtained from “Clinical electroencephalography (with elements of epileptology)” [1]. The book systematically presents the neurophysiological and biophysical fundamentals of EEG. The methodology of EEG analysis and electroencephalographic semiotics are given. In Appendix A, “Practical application of computer electroencephalography” [1], the author describes in detail a number of experiments, including the study of evoked potentials of functional samples which are used to prepare the experiment for depression of the exaltation of alpha rhythm.

The research [12] describes the use of brain physiological signals to control a robotic system. Authors create brain machine interface (BMI) using the steady state visual evoked potentials technique (SSVEP). Instead of using EEG error potential identification (ErrP), authors instructed the users to explicit misclassifications using one of the two following extra-brain activities: briefly clenching teeth or closing the eyes. The experiments, conducted on three healthy subjects, show that these two extra-brain activities are detectable by EEG time-frequency analysis and in less than one second if the user is focused.

## 3. Materials and Methods 

### 3.1. Use of Open Sources in the Development of BCI 

The main objective was that BCI should be able to recognize brain activity. Artificial neural network (ANN) was used for this purpose. It is necessary to choose from the list of available libraries. Preference was given to the Accord.NET Framework. The Accord.NET Framework is a .NET machine learning framework combined with audio and image processing libraries completely written in C#. It is a complete framework for building production-grade computer vision, computer audition, signal processing and statistics applications, even for commercial use. A comprehensive set of sample applications provide a fast start to getting up and running quickly, and extensive documentation and wiki helps fill in the details. The library is licensed by GNU Lesser Public License v2.1.

**Figure 4:**
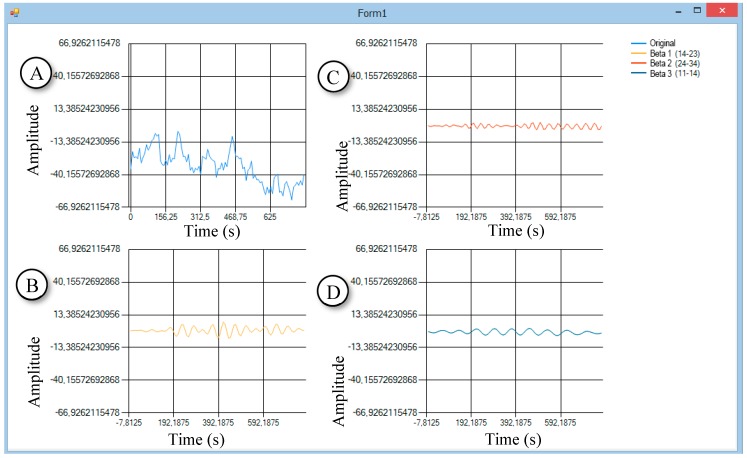
One of the first versions of the software. It works with static data (EEG recording files) using Butterworth finite impulse response (FIR) filters. Light blue (**A**)—raw EEG. Yellow (**B**)—beta 1 waves. Red (**C**)—beta 2 waves. Dark blue (**D**)—beta 0 waves.

The second step in the development of software was around the reception and use of the human EEG. It was necessary to solve the problem with the separation of the EEG into its constituent rhythms/waves. Development of the software mainly relies on materials on Butterworth filters with finite impulse response (FIR) [13]. The GUI of the software is shown in Figure 4. 

The software with several libraries for filtering was tested on the third step; the Math.NET Filtering library was chosen. The library is licensed under the MIT/X11 License. Filtering aims to provide a toolkit for digital signal processing, offering an infrastructure for designing digital filters and applying those filters to data streams using data converters as well as digital signal generators. For testing, samples were used as a data source from Physio Net [14]. The library implements the Butterworth filter.

### 3.2. Hardware Configuration for the Bitronics

The Bitronics “Young Neuromodelist” Design Set is the first educational set for the study of bio-signals. The kit included all of the necessary hardware for the obtaining the EEG. In this study, the following equipment was used:

- Electrodes on the bandana-bond,
- The reference electrode in the form of a clip connected to the ear,
- Electrodes connected to the EEG module from Bitronics, which acts as a differential amplifier.

**Figure 5:**
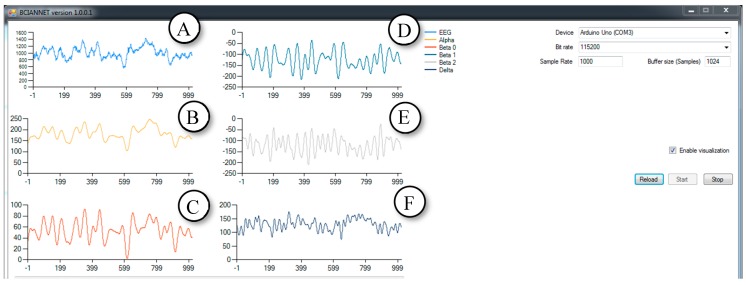
Improved version of the software which works similarly to the previous one but is an updated version that takes data from Arduino. Light blue (**A**)—raw EEG. Yellow (**B**)—alpha waves. Red(**C**)—beta 0 waves. Cyan (**D**)—beta 1 waves. Grey (**E**)—beta 2 waves. Dark Blue (**F**)—delta waves.

**Figure 6:**
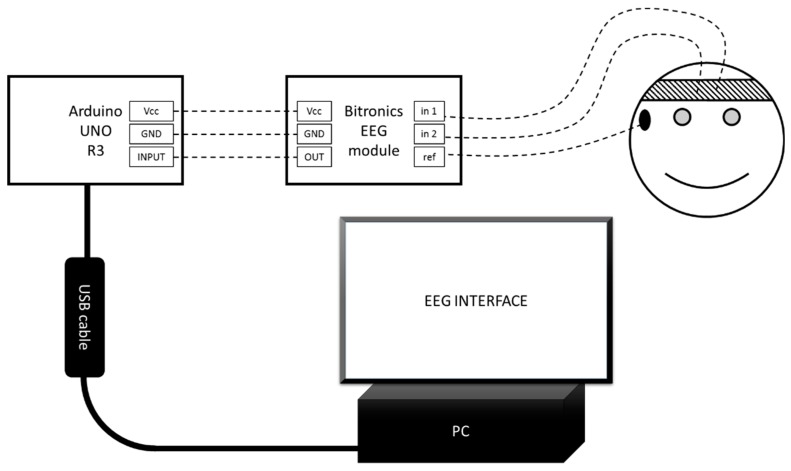
Schematic diagram of the brain computer interface (BCI).

After this, an interface was developed. The graphical user interface (GUI) is depicted in Figure 5. The hardware consists of the modules EEG Bitronics, Arduino and a computer (Figure 6).

Figure 6 above depicts the PC-any personal computer with Universal Serial Bus (USB) and Windows Operating System (OS). EEG INTERFACE is a Software which was developed in this research. The USB cable was a standard USB A-B cable. Arduino UNO R3 is a microcontroller board based on the ATmega328P with an option of USB connection [15]. Bitronics EEG module is an EEG module from the Bitronics kit, Vcc are power pins, and GND are ground pins. In1 and in2 are pins for signal electrodes. Ref is a pin for reference electrode. OUT is an EEG module’s analogue output pin. INPUT is an Arduino’s analogue input pin. The smiley illustrates a subject (human) with connected electrodes. The bandana is signal electrodes. The ear clip is a reference electrode.

The signal appearing on the cerebral cortex is amplified and shifted in the EEG module from Bitronics. On the module, there are control knobs for the amplification and noise reduction of the signal. It is important, whilst working to configure the module, not to overload the signal (the voltage should not exceed the reference voltage on the Analog-to-digital converter (ADC)). In the case of Arduino, it is 5 V from the power supply via USB. The output signal is read using an analogue digital converter in Arduino. The signal is encoded and sent to the computer via a virtual serial COM port (communication port). There is a software that receives data and the processing is running on the computer at this time.

There is a standard software and a standard sketch included in the set of Bitronics for sensor monitoring and for Arduino. The sketch periodically takes readings from two analogue ports and sends the value to the computer in the form of lines, adding “A0” and “A1” to them, depending on which analogue port’s voltage value is sent. The software, along with sketch for Arduino were both written by the authors. In contrast to the Bitronics sketch, in the authors’ sketch, the information is transmitted as bit-by-bit numbers.

The written software was made by the authors for the PC. It buffers the data, produces filtering and determines the amplitude of the EEG rhythms in the signal. The software program BCIANNET is developed by using .Net Framework and C# language’s libraries. As a result, the authors created software called BCIANNET v2.0.0.1 which works as follows:

- There is an event handler [16] attached to the data arrival event to transfer the bits of information into numbers and fill the buffer, which stores 1000 samples. This corresponds to 10 seconds with a sampling rate of 100 transforms per second.
- A Butterworth FIR filter filters the signal 60 times per second. In this way, we get the components of the signal—the rhythms of the brain. Then, the average amplitude of the components is calculated.
- Graphical interface updates separately, 60 times per second. The program updates the diagrams and shows values of the variables on the screen.
- During the experiments, the program also buffers the received values of the EEG rhythms and uses them to train the ANN. When the training of the neural network ends, the amplitudes are used as inputs to the neural network.

Twelve human subjects took part in these experiments. The mean age was 26.4 years, the minimum age was 11 years and the maximum age was 53 years. Most of the subjects were students and their health status was good. In the experiment, seven male and five female subjects were involved. Individual subjects’ descriptions are shown in Table 1.

### 3.3. The First Series of Experiments

The software development started with using the Bitronics kit. In the beginning, the software developed by the authors was used to determine the purpose of the pins, potentiometers and eventually to obtain an online stream of data suitable for later analysis. 

**Figure 7:**
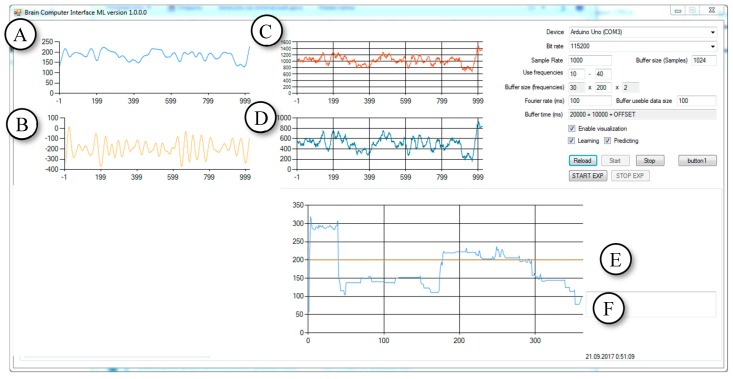
The Brain Computer Interface ML Version 1.0.0.0 program evaluates the amplitude of the alpha rhythm. (Experiment eyes open (EO)/eyes closed (EC). Light blue (**A**)—alpha waves. Yellow (**B**)—beta 1 waves. Red (**C**) and cyan (**D**) are raw EEG in a different scale. Yellow (**E**)—graph displaying the status of the experiment in accordance with the scale of the A/EEG ratio graph. Light blue (**F**)—alpha/raw EGG amplitude ratio (multiplied by 1000). (Opened eyes—value is 100, closed eyes—value is 200).

In order to improve the application, the filtering algorithm was prepared. Then, the authors proceeded to one of the most difficult parts—the implementation of the information transferring from Arduino to the computer. The event handler [16] was attached to the data arrival event. This event caches the received values into the buffer. An important part is also the possibility of setting parameters, such as the sample rate for the COM-port and sampling frequency (important for filtering). The first series of experiments were conducted with subject #1. The object was the Brain-Computer Interface Machine Learning (ML) Version 1.0.0.0 program that has been developed in this study. The updated version of the software is shown in Figure 7.

The purpose of the experiments was to test and confirm the performance of the software to read the EEG data of the subjects by using the connected external devices and the performance to distinguish between changes in the EEG caused by the actions of the different subjects. The software was developed in such a way that after receiving the data stream, it separates the alpha-rhythm from the EEG signals by means of the FIR-filter and finds the average amplitude. After that, the program finds the ratio of the amplitude of the alpha rhythm to the total amplitude. The graph displays the probability as a time function, which is shown in Figure 7F. The higher the ratio of amplitudes shown in Figure 7F, the more likely it is that the subject closes their eyes. In this version of the software, statistical tools are not implemented. The subject visually analyzes the output, and the program serves to confirm the ability to detect differences in the flow of incoming data. 

As a change in the condition of the subjects, the alpha rhythm is considered. The subject closes or opens their eyes (eyes open (EO) or eyes closed (EC)) on the automatic generated program command (short beep signal—”beep”) in response to the sound marker (low tone of 1000 HZ—eyes closed, high tone 1500 HZ—eyes open). A similar experiment on reactivity with sample analysis (type EO/EC) is described in detail in [1] and serves to identify the reaction according to the type of EO/EC in the observed subjects. It is known that with closed eyes, alpha rhythms increase. In the context of the current experiment, the program must identify these amplitude changes by reflecting on the current graph. It should be noted that this experiment serves to identify the differences in incoming data and waveforms, and does not perform medical tasks (diagnosis). The program’s window has a small window for the diagram of sound markers. This graph is displayed together with the graph obtained by the program of analyzing the amplitude deviations. The synchronization (correlation) of these two graphs in time means the ability to distinguish these amplitude deviations. The program’s operation and the actions of the subjects were recorded using the Icecream Screen Recorder program [17] and a web-camera connected to the computer. The duration of each experiment was 60 s. After that, the received records were analyzed and the test results were recorded in the report. Such a structure of the program makes it possible to combine in one person the functions of each subject. A series of eight such experiments were conducted at different times of the day. As per our understanding, we considered and took into account lighting effect as an additional factor, as this can also affect the nature of the alpha rhythm.

At the end of the first series of experiments, a new version of the software was created based on the tested program. Changes primarily concerned the analysis of amplitude oscillations, but the most important changes concerned the method of analyzing the ratio between the amplitude oscillations of different rhythms. In this series of experiments, the machine learning method based on the operation of the perceptron neural network was applied. Another significant change concerned the statistical recording of the results of the work of the artificial neural network (ANN), which made it possible to evaluate its effectiveness. The researcher has updated this program with four algorithms, given the fact that there are several different methods of machine learning. 

### 3.4. The Second Series of Experiments

In the second series of the experiments, the author tested the updated software called BCIANNET v.2.0.0.1. It was conditionally divided into four sub-series. In each subseries, two or three subjects took part. In each subseries one machine learning algorithm was used. The electrodes were configured on the head of the subject, the operation of the devices was checked and all of the interface components were also checked. The researcher started the experiment with the recording of the data, in analogy with the first series of experiments. As in the first series, the reference for the subject was sound signals of high and low frequencies.

**Figure 8:**
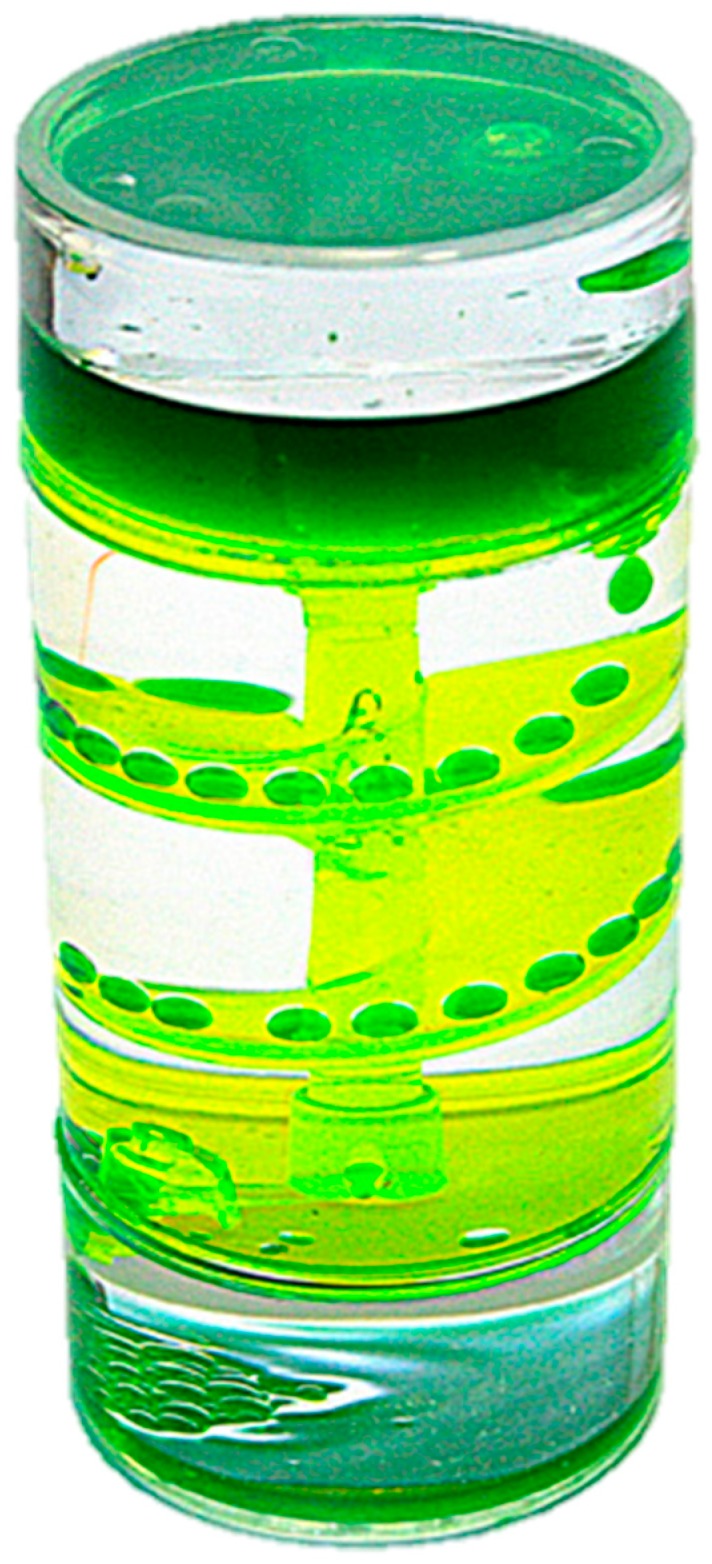
Anti-stress toy.

**Figure 9:**
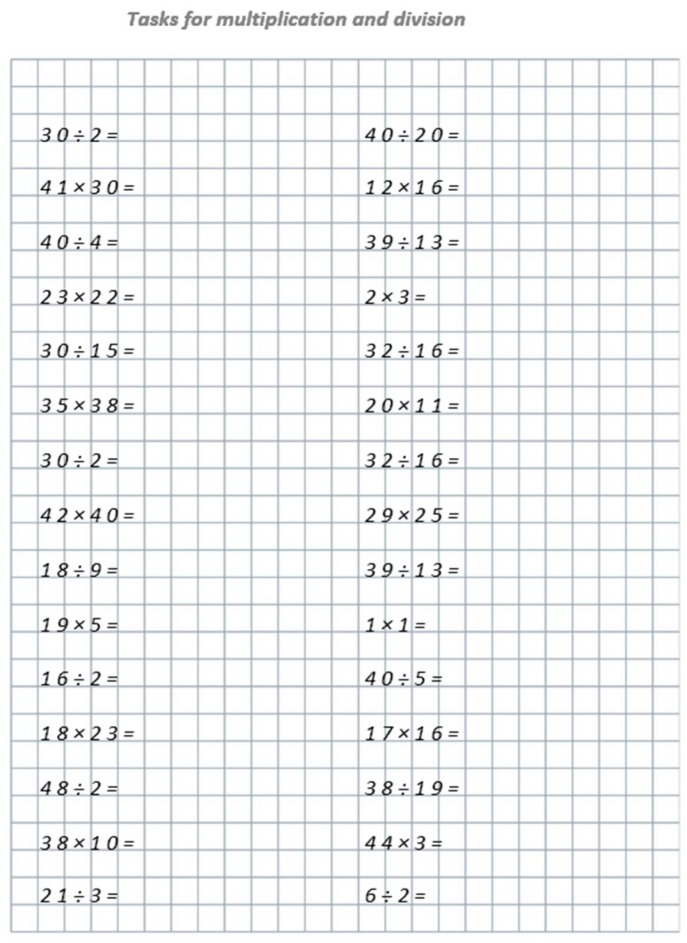
Examples of calculating tasks.

The subject’s task was to independently influence the EEG indications of his own brain (biofeedback). To do this, the subject was given tasks to stimulate changes in brain activity. The first task was to look at the anti-stress toy, which is shown in Figure 8. The second was to calculate the simple calculation problems from the computer screen. The simple calculation problems are given in Figure 9.

The task of the ANN was to determine the type of activity by analyzing the incoming data from the configured electrodes. The interface program, as it did in the first series of experiments, demonstrated amplitude deviations, so that the subject could see the result of his own activity analyzed by the ANN.

**Figure 10:**
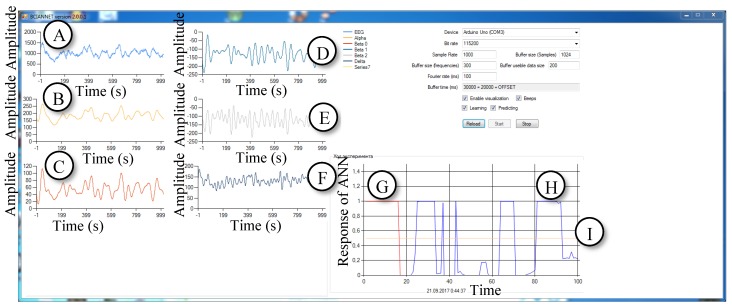
The final version of the software BCIANNET v 2.0.0.1. It assesses the state of a person using the artificial neural network (ANN). Light blue (**A**)—raw EGG. Yellow (**B**)—alpha waves. Red(**C**)—beta 0 waves. Cyan (**D**) are beta 1 waves. Grey (**E**)—beta 2 waves. Dark blue (**F**)—delta waves. Red (**G**)—graph displaying the status of the experiment (1 or 0). Blue (**H**)—graph indicating neural network predictions. Yellow (**I**)—mean value from experiment states from array of data, which is used as an output dataset for neural network learning (0.5 value means that everything is normal).

This created the conditions for the emergence of biofeedback. Unlike the first version of the program, statistical data was collected in the upgraded final version of the program, which is depicted in Figure 10. It generated a log file from two rows of numbers (zeros and ones). The first row of the log file indicates the sound markers generated by the final program that serves for the subject to change actions. A high tone corresponds to zero, a low tone corresponds to one. In the second row, zero means that the program detected the background activity, and one means that the neural network detected a deviation in the amplitude ratio provoked by the subject.

After the experiment, the researcher saved the data protocol and log file for its subsequent analysis. In the acquired dataset, the first 60 seconds and the last 10 seconds of the acquired data was not used because the first 60 seconds were considered as the time when the neural network was being trained (there was an accumulation of data for the initial training of the neural network). In addition, subjects should be able to adapt to their role and work with the interface. The last 10 seconds were not considered in order to reduce the most probable reaction of the subject to the expectation of nearing the completion of the experiment. Obviously, such a structure of the experiment allowed us to determine the effectiveness of learning and recognizing through the ANN, as well as the ability to consciously control a computer exclusively by brain activity.

The BCIANNET v.2.0.0.1 software used four types of machine learning algorithms—error correction algorithm [18], backpropagation [19], Rprop (resilient backpropagation) [20] and the Levenberg–Marquardt algorithm [21]. At the stage of compilation, it was decided which type was going to be used to collect the data from subject. All of these algorithms work with perceptron ANN. The structure of the neural network depends on the learning method. The error correction algorithm is the easiest and can teach only a single-layer perceptron with a structure of 5-1. In other cases, the author uses networks with a large number of layers with a structure of 5-6-6-1. The inputs were amplitudes of brain waveforms, while output was state of brain (thinking or resting). ANN was implemented using the Accrod.Neuro library (part of the Accord.Net framework).

## 4. Results

### 4.1. Results of the First Series of Experiments 

Eight tests were conducted in the first series of the experiment. The authors controlled the reaction EO/EC (subject closes eyes or opens eyes) of the subjects. A series of experiments was carried out at different lighting in case the lighting affected the tests results. The lighting measurements were carried out with the help of the Velleman DVM1300 Digital Light Meter. A series of experiments was carried out with the first subject. In total, eight experiments were conducted at different times. The purpose of the first series of experiments was to test the configuration and setting up of the devices, such as the Bitronics EEG module, Arduino, computer and developed software BCIANNET v1.0.0.1, to see the reaction of EO/EC and analyze the results. The authors observed the response EO/EC taking into account the graph of the amplitude of the alpha-rhythm.

The date, time, conditions and results of the experiments of the first series are presented in Table 2.

It can be seen from the table that in all of the conducted experiments, an EO/EC reaction was present. During the experiments, the authors found a correlation between the amplitude of the alpha-rhythm and the state of the eyes. The goal was to confirm the effectiveness of the Brain-Computer-Interface ML Version 1.0.0.0 by its ability to recognize changes in the rhythm of the EEG. Based on our understanding and shown by the study, light does not have a large impact on the results.

### 4.2. Results of the Second Series of Experiments 

As soon as the authors received the results of the first series of experiments, they were convinced of the operability of the hardware and the software and developed the next version of the software. The authors released an updated version of the software BCIANNET v.2.0.0.1 and proceeded with the second experiment. 

Four tests were conducted. In the first test, the authors found out that it was better to make a 10-second interval between functional tests in further experiments. The beep concept, which involved the use of a beep to indicate the state of brain activity, was introduced in the study. 

BCIANNET v.2.0.0.1 saves the log file. There are two columns to indicate the expected data and the generated data by the neural network. As has already been mentioned, the beginning and the end of the data were not used for the sake of obtaining a more reliable result. It was decided that in each sample, 400 values would be taken, which corresponds to 40 s of recording. Two experiments were performed for each subject in each subseries of experiments. 

As mentioned above, BCIANNET v.2.0.0.1 software was used in the second experiment with the application of filtering, amplitude calculation and ANN. There were 12 subjects, but some experiments were considered invalid. In some cases, subjects saw themselves on the screen, started laughing and could not concentrate. Special samples of data were taken and the obtained diagrams were analyzed. The evaluation was performed by selecting 400 rows in the middle of the log file and calculating the percentage of matches. The results are shown in Table 3.

As shown in Table 3, 14 experiments were done in the first subseries of experiments, 13 experiments in the second and third subseries and 12 experiments were done in last subseries. In the beginning, four experiments were conducted in order to find out the best time for changing the state (5 or 10 s intervals). The test results showed that with the intervals of 10 seconds, the software gives better results. All subsequent series and the remainder of the experiments of the first series were conducted with a 10-second interval. During the experiments with the third subject, problems arose in that the subject #3 laughed seeing his picture during the experiment. Because of this, the experiments were not sufficiently clear and it was decided to take into consideration only one experiment (in the second subseries). 

**Figure 11:**
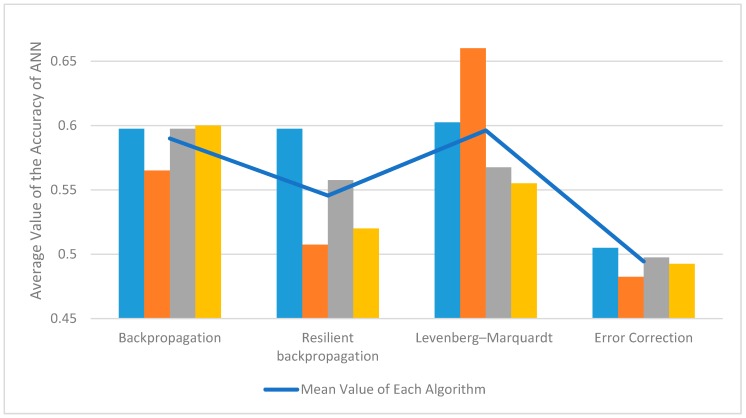
The average value of the accuracy of machine learning algorithms taking into account only four experiments in each series. Blue—subject 1, orange—subject 2, gray—subject 3, yellow—subject 4.

Figure 11 shows perhaps more equal results for each learning algorithm; two subjects were used. Two experiments were conducted for each subject and four experiments on each algorithm. The mean of the graph in Figure 11 indicates the mean accuracy values of learning algorithms.

## 5. Conclusions

The purpose of this research study was to explore the possibility of developing a brain-computer interface (BCI). The main conclusion is that the interface consisting of EEG Bitronics, Arduino and a computer is able to recognize brain activity and separate important rhythms such as alpha, beta, gamma and delta from other EEG signals with the help of BCIANNET. Based on the results of the series of experiments, it can be stated that the algorithm Levenberg–Marquardt was found to be the best among the algorithms tested. The data obtained during the experiments allows us to confirm the effectiveness of the Levenberg–Marquardt algorithm as applied to the development of BCI, and that the latest version of the software developed by the authors is an effective tool for research in the field of BCI.

The experience gained in the study allows us to plan and implement a further study which will focus on one of the following directions and ensure the further evolution of the technology:

- Providing more noise immunity, finding a way to reduce the influence of external factors, reducing the negative effect of the inability of the subject to concentrate on the experiment (spontaneous reactions should be envisaged and maintain the effectiveness of the interface).
- The study of variations associated with the hardware, improving ergonomics.
- Study the possibility of increasing the number of electrodes involved without compromising ergonomics.
- Expanding the variability of data entry (not just 0 or 1).
- Medical direction, including the study of the ability and inclination of the subject to use this interface.

The main result of the research can be considered for the development and testing of the interface, which includes hardware and software. Following the specification (see the Appendix A) and using the developed software, it is possible to continue researching available algorithms and libraries of ANN. The developed interface allows the determination of the most promising paths for further development with the help of simple testing. 

## Figures and Tables

**Table 1:** Statistics of subjects who took part in experiment.

**No.**	**Gender**	**Age**	**Spent Hours**	**Series of Experiments**
1	Male	15	40	Testing and carrying out of two series of experiments
2	Male	44	5	Second series of experiments
3	Male	15	1	Second series of experiments
4	Male	17	1	Second series of experiments
5	Male	17	1	Second series of experiments
6	Male	17	1	Second series of experiments
7	Female	12	1	Second series of experiments
8	Female	44	1	Second series of experiments
9	Female	24	1	Second series of experiments
10	Female	48	1	Second series of experiments
11	Male	53	1	Second series of experiments
12	Female	11	1	Second series of experiments
**Summary**				
Total # of Subjects:				12
Mean Age:				26.4
Minimum Age:				11
Maximum Age:				53
Male:				7
Female:				5

**Table 2:** Experiment 1.

No.	Date	Time	Lighting (LUX)	Presence of EO/EC Reaction
1	24.08.2017	11:30	260	Yes
2	24.08.2017	11:36	20	Yes
3	24.08.2017	22:30	113	Yes
4	24.08.2017	22:35	9	Yes
5	25.08.2017	11:25	220	Yes
6	25.08.2017	11:30	40	Yes
7	26.08.2017	23:57	146	Yes
8	27.08.2017	0:08	8	Yes

**Table 3:** Results of the second experiment.

Date	Time	Subject	Algorithm Name	Beep	Accuracy
03.09.2017	15:10	1	Backpropagation	10	0,5975
03.09.2017	17:44	1	Backpropagation	10	0,565
03.09.2017	18:11	1	Backpropagation	5	0,4675
03.09.2017	18:20	1	Backpropagation	5	0,56
03.09.2017	18:38	2	Backpropagation	10	0,5975
03.09.2017	18:48	2	Backpropagation	10	0,6
14.08.2018	10:06	4	Backpropagation	10	0,54
17.08.2018	13:03	5	Backpropagation	10	0,511
17.08.2018	13:28	6	Backpropagation	10	0,52
17.08.2018	13:56	7	Backpropagation	10	0,574
17.08.2018	20:13	8	Backpropagation	10	0,5419
19.08.2018	12:33	9	Backpropagation	10	0,5839
20.08.2018	18:15	10	Backpropagation	10	0,515
20.08.2018	18:45	11	Backpropagation	10	0,565
04.09.2017	22:32	1	Resilient backpropagation	10	0,5975
04.09.2017	22:40	1	Resilient backpropagation	10	0,5075
05.09.2017	22:09	2	Resilient backpropagation	10	0,5575
05.09.2017	22:17	2	Resilient backpropagation	10	0,52
04.09.2017	23:42	3	Resilient backpropagation	10	0,5575
14.08.2018	10:10	4	Resilient backpropagation	10	0,535
17.08.2018	13:15	5	Resilient backpropagation	10	0,524
17.08.2018	13:35	6	Resilient backpropagation	10	0,521
17.08.2018	14:01	7	Resilient backpropagation	10	0,56
17.08.2018	20:20	8	Resilient backpropagation	10	0,5575
19.08.2018	12:41	9	Resilient backpropagation	10	0,535
20.08.2018	18:20	10	Resilient backpropagation	10	0,525
20.08.2018	18:50	11	Resilient backpropagation	10	0,572
04.09.2017	22:52	1	Levenberg–Marquardt	10	0,6025
04.09.2017	23:04	1	Levenberg–Marquardt	10	0,66
05.09.2017	22:25	2	Levenberg–Marquardt	10	0,5675
05.09.2017	22:36	2	Levenberg–Marquardt	10	0,555
14.08.2018	10:03	4	Levenberg–Marquardt	10	0,544
17.08.2018	12:57	5	Levenberg–Marquardt	10	0,521
17.08.2018	13:20	6	Levenberg–Marquardt	10	0,56
17.08.2018	13:48	7	Levenberg–Marquardt	10	0,5875
17.08.2018	20:07	8	Levenberg–Marquardt	10	0,596
19.08.2018	12:23	9	Levenberg–Marquardt	10	0,55
20.08.2018	18:10	10	Levenberg–Marquardt	10	0,531
20.08.2018	18:37	11	Levenberg–Marquardt	10	0,601
20.08.2018	19:07	12	Levenberg–Marquardt	10	0,5503
05.09.2017	21:03	1	Error Correction	10	0,505
05.09.2017	21:32	1	Error Correction	10	0,4825
06.09.2017	22:00	2	Error Correction	10	0,4975
06.09.2017	22:05	2	Error Correction	10	0,4925
14.08.2018	10:15	4	Error Correction	10	0,505
17.08.2018	13:24	5	Error Correction	10	0,51
17.08.2018	13:40	6	Error Correction	10	0,4975
17.08.2018	14:14	7	Error Correction	10	0,505
17.08.2018	20:27	8	Error Correction	10	0,5495
19.08.2018	12:50	9	Error Correction	10	0,521
20.08.2018	18:26	10	Error Correction	10	0,4984
20.08.2018	18:00	11	Error Correction	10	0,521

## Appendix A

**Specification**

**Name**	**Count**
Arduino Uno R3	1
Bitronics EEG module	1
Cable USB (A-B)	1
EEG referent clips	1
EEG bandana electrodes from Bitronics	1
PC with USB and Windows 7+ monitor	1
Arduino Sketch	Programm (by author)
BCIANNET	Programm (by author)
Breadboard	1
Connecting wires	3
